# Energy status regulates levels of the RAR/RXR ligand 9-*cis*-retinoic acid in mammalian tissues: Glucose reduces its synthesis in β-cells

**DOI:** 10.1016/j.jbc.2023.105255

**Published:** 2023-09-14

**Authors:** Hong Sik Yoo, Kristin Obrochta Moss, Michael A. Cockrum, Wonsik Woo, Joseph L. Napoli

**Affiliations:** Department of Nutritional Sciences and Toxicology, Graduate Program in Metabolic Biology, University of California, Berkeley, Berkeley, California, USA

**Keywords:** autophagy, FoxO1, insulin, liquid chromatography, mass spectrometry, nuclear receptors, pancreas, RAR, Rdh5, retinoic acid, RXR, transcription

## Abstract

9-*cis*-retinoic acid (9cRA) binds retinoic acid receptors (RAR) and retinoid X receptors (RXR) with nanomolar affinities, in contrast to all-*trans*-retinoic acid (atRA), which binds only RAR with nanomolar affinities. RXR heterodimerize with type II nuclear receptors, including RAR, to regulate a vast gene array. Despite much effort, 9cRA has not been identified as an endogenous retinoid, other than in pancreas. By revising tissue analysis methods, 9cRA quantification by liquid chromatography-tandem mass spectrometry becomes possible in all mouse tissues analyzed. 9cRA occurs in concentrations similar to or greater than atRA. Fasting increases 9cRA in white and brown adipose, brain and pancreas, while increasing atRA in white adipose, liver and pancreas. 9cRA supports FoxO1 actions in pancreas β-cells and counteracts glucose actions that lead to glucotoxicity; in part by inducing *Atg7* mRNA, which encodes the key enzyme essential for autophagy. Glucose suppresses 9cRA biosynthesis in the β-cell lines 832/13 and MIN6. Glucose reduces 9cRA biosynthesis in 832/13 cells by inhibiting *Rdh5* transcription, unconnected to insulin, through cAMP and Akt, and inhibiting FoxO1. Through adapting tissue specifically to fasting, 9cRA would act independent of atRA. Widespread occurrence of 9cRA *in vivo*, and its self-sufficient adaptation to energy status, provides new perspectives into regulation of energy balance, attenuation of insulin and glucose actions, regulation of type II nuclear receptors, and retinoid biology.

The parent retinoid, retinol, produces multiple metabolites, with all-*trans*-retinoic acid (atRA) functioning as a major autacoid (1, 2). atRA activates retinoic acid (RA) receptors (RAR), but also acts through noncanonical mechanisms (3, 4). An atRA isomer, 9-*cis*-retinoic acid (9cRA) binds with high affinity to both RAR and retinoid X receptors (RXR) *in vitro* (5). 9cRA occurring in tissue concentrations similar to atRA would verify presence of an autacoid with potentially more extensive regulatory impact than atRA. Since its identification as a high-affinity RAR and RXR ligand, however, the *in vivo* occurrence of 9cRA has been controversial (5, 6, 7). Multiple searches failed to demonstrate 9cRA as an endogenous tissue retinoid (8). These included liquid chromatography-tandem mass spectrometry (LC/MS/MS) assays validated for tissue analyses of RA isomers (9, 10, 11). Inability to quantify 9cRA *in vivo* initiated searches for an endogenous RXR ligand. Phytanic acid, docosahexaenoic acid, and several polyunsaturated fatty acids were proposed as RXR ligands, but each requires 4 to >10 μM to activate RXR, that is, several orders of magnitude less potent than 9cRA (12, 13, 14, 15). None attains *in vivo* concentrations as free acids capable of activating RXR, and none activate RAR. A more recent conclusion that 9-*cis*-13,14-dihydroretinoic acid serves as a physiological RXR ligand faces several drawbacks (16). It does not bind RAR at all, and its more active R-enantiomer has no affinity for RXR at 100 nM; at 10 μM it exerts only ∼40% of 9cRA’s binding affinity. Its affinity was estimated by a reporter assay with an RXR ligand-binding domain transfected into COS cells, which did not achieve saturation. These limitations pose substantial concern about the relevance of 9-*cis*-13,14-dihydroretinoic acid as an RXR ligand, especially if 9cRA were present in tissues.

The question of 9cRA as an endogenous retinoid was partially resolved by its quantitation in pancreas in concentrations similar to or higher than atRA (17, 18). Pancreas 9cRA relates inversely to serum glucose and insulin in mice, and induces glucose intolerance during glucose-tolerance tests. 9cRA in nanomolar concentrations represses glucose-stimulated insulin secretion (GSIS) in pancreatic β-cells and in mouse islets through inhibiting glucose uptake by Glut2 and glucose phosphorylation by glucokinase (Gck), posttranscriptionally. 9cRA also suppresses *Pdx-1* and *HNF4α* mRNA transcriptionally. Pdx-1 induces *Gck*, whereas HNF4α induces *Glut2*, and both stimulate insulin release through enhancing mitochondrial metabolism (19, 20, 21). Defects in *GK*, *Pdx-1*, and *HNF4* underlie maturity onset diabetes of the young (MODY1, 2, and 4). 9cRA decreases in mouse models of reduced β-cell content, such as heterozygous Akita mice and streptozotocin-treated mice. In contrast, 9cRA rises to abnormally high levels in glucose-intolerant mice with β-cell hypertrophy, including mice with diet-induced obesity, as well as *ob*/*ob* and *db*/*db* mice. These data established 9cRA as a pancreatic autacoid that modifies GSIS, and beg the question whether 9cRA occurs endogenously in other tissues.

Widespread occurrence of 9cRA *in vivo* would inform its therapeutic applications. As the drug alitretinoin, 9cRA exerts ameliorating effects on skin cancers, such as cutaneous T-cell lymphoma and Kaposi’s sarcoma, therapy-resistant lichen planus, and chronic hand eczema (22, 23, 24). 9cRA also has been evaluated for chemoprevention and chemotherapy, because it promotes neuroblastoma cell differentiation, inhibits growth of the human myeloid leukemia cell line HL-60 with greater potency than atRA, induces differentiation of isolated human leukemia cells, and inhibits growth of the estrogen receptor-positive breast cancer cell line MCF7 (25, 26).

Potential for new therapeutic agents has spawned synthesis and evaluation of 9cRA analogs that bind specifically with RXR, known as rexinoids (27, 28). The Food and Drug Administration has approved one of these, Targretin (bexarotene), for treating cutaneous T cell lymphoma (29, 30). Rexinoids also decrease hyperglycemia and hyperinsulinemia by improving skeletal muscle insulin sensitivity in mouse models of obesity and noninsulin–dependent diabetes (31, 32, 33, 34, 35). Although rexinoids may prove useful as insulin sensitizers and to reduce obesity, they also present with deleterious side effects. These include suppressing thyroid hormone action, increasing serum triglycerides and inducing hepatomegaly (34). The diverse actions of rexinoids likely result from RXR heterodimerization with type II nuclear receptors, including FXR, LXR, PPAR, PXR, RAR, THR, and VDR (36). Understanding locations and regulation of 9cRA in tissues should provide data useful to evaluating clinical application of rexinoids.

The present work aimed to determine whether 9cRA occurs in tissues other than pancreas, and if so, to provide insight into its presence vis-à-vis atRA, and regulation of its biosynthesis. We report widespread tissue occurrence of 9cRA in concentrations similar to atRA. 9cRA and atRA vary tissue specifically and independently in tissues with fasting *versus* refeeding. In β-cells, glucose reduces Rdh5-catalyzed 9cRA biosynthesis, accounting for decreased 9cRA during the fed state. Variation of 9cRA with energy status complements actions of type II nuclear receptors, which regulate genes that modify energy metabolism, among other functions.

## Results

### Prevention of RA isomerization

**Figure 1:**
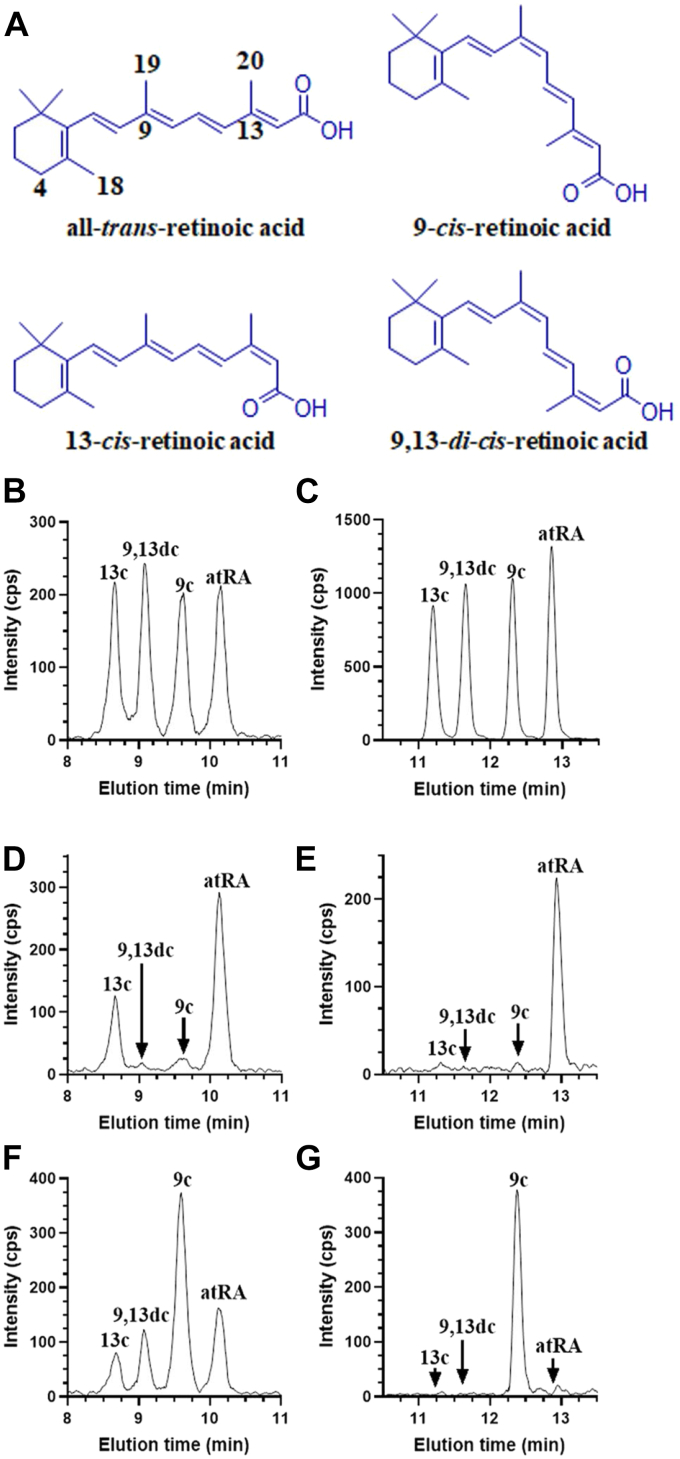
**Comparison of RA resolution and isomerization during extraction.***A*, structures of RA isomers. Standards of RA isomers (100 fmol each) were resolved by either (*B*), original LC conditions or (*C*), revised LC conditions. *D*, atRA and (*F*), 9cRA standards in saline/ethanol were extracted and resolved by the original LC conditions. *E*, atRA and (*G*), 9cRA standards in methanol were extracted and resolved by the revised LC conditions. Analyses were done by LC/MS/MS using multiple reaction monitoring with a triple quadrupole mass spectrometer, which first isolated the molecular ion at *m/z* 301 [M + H]^+^ in Q1 and then quantified its product ion at *m/z* 205 in Q3. 9cRA, 9-*cis*-retinoic acid; atRA, all-*trans*-retinoic acid; LC, liquid chromatography; LC/MS/MS, LC-tandem mass spectrometry; RA, retinoic acid.

**Figure 2:**
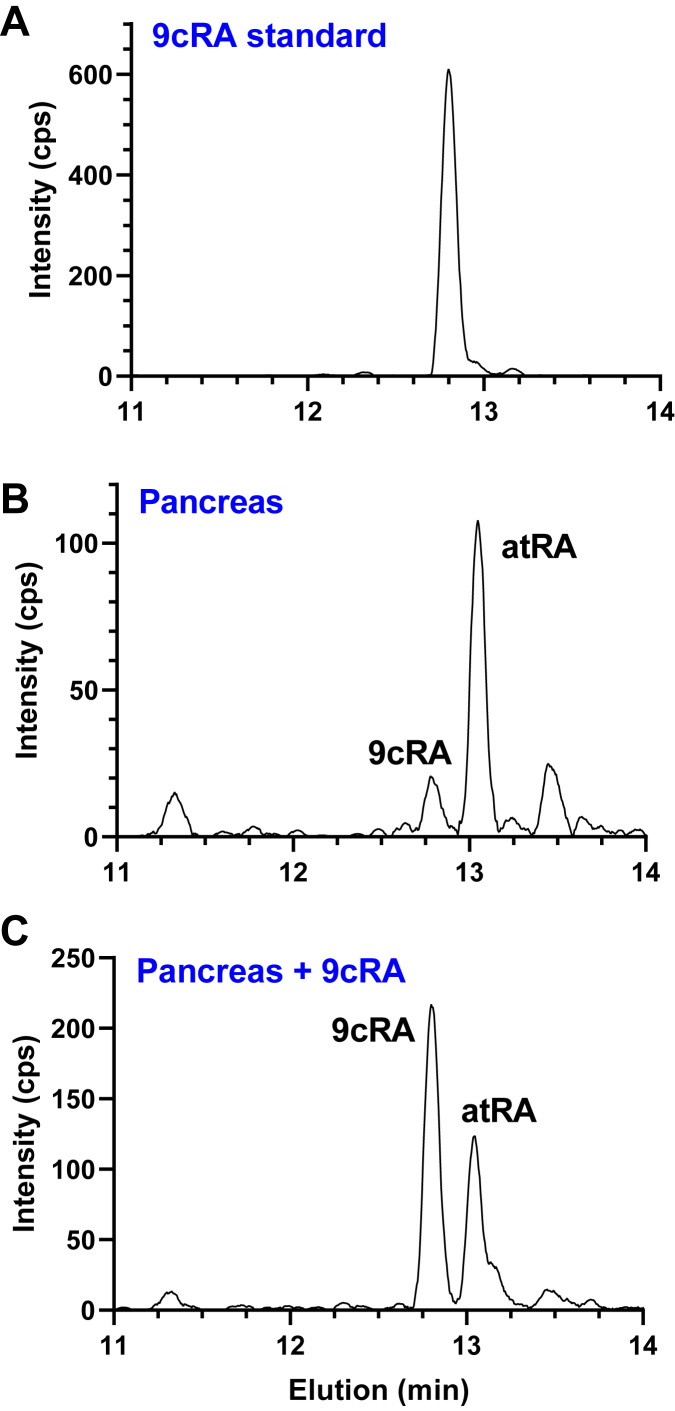
**Recovery of 9cRA from pancreas without isomerization.***A*, one microliter of a 100 nM solution of 9cRA in methanol (100 fmol) was injected into LC/MS/MS. *B*, a pancreas from a male mouse was homogenized in 1.5 ml methanol. Half of the homogenate was analyzed using the revised protocol. *C*, ten microliters of a 100 nM solution of 9cRA was added to the remaining half of the pancreas homogenate and analyzed using the revised protocol. Recovery was 96%. 9cRA, 9-*cis*-retinoic acid; LC/MS/MS, LC-tandem mass spectrometry.

Skepticism about 9cRA as an autacoid has been fostered by failure to identify it in tissues using analyses optimized for quantifying atRA. To address this, we revised recovery and liquid chromatography (LC) protocols for quantifying RA isomers in serum and tissues by returning to tissue homogenization with methanol, rather than with saline/ethanol, and modifying LC elution to improve resolution (37). The original LC mobile phase resolved four RA isomers (Fig. 1). The revised LC protocol improved resolution (Fig. 1, *B* and *C*). The original saline/ethanol homogenization resulted in atRA isomerizing partially into 13cRA (Fig. 1*D*) and 9cRA isomerizing partially into atRA (9,13-di-*cis*-RA (9,13dcRA) and 13-*cis*-RA (13cRA) (Fig. 1*F*). In contrast, methanol-based homogenization maintained the integrity of extracted atRA and 9cRA (Fig. 1, *E* and *G*). To verify reliability of the methanol-based procedure, we added 9cRA to a pancreas homogenate, extracted the sample, and compared it to a sample without added 9cRA (Fig. 2). The methanol-based procedure recovered 9cRA without isomerization.

### Full-scan mass spectrum of endogenous 9cRA

**Figure 3:**
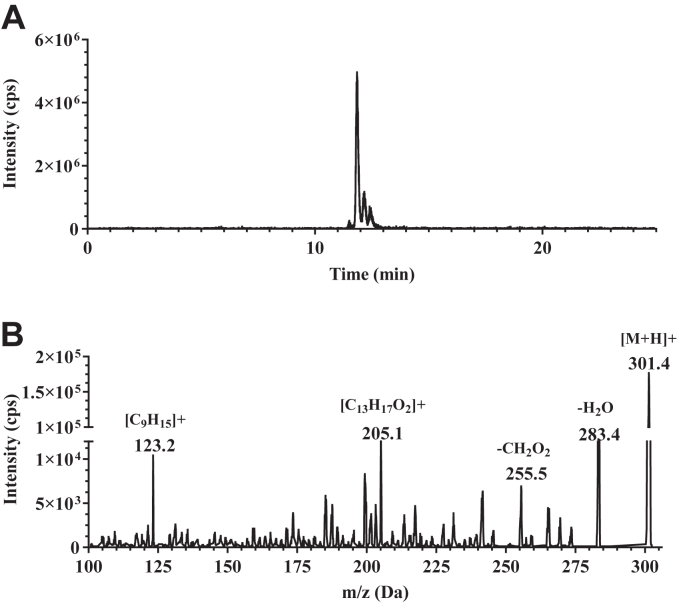
**Mass spectrum of endogenous 9cRA.** Pancreata from 46 mice were pooled, homogenized in methanol, and applied to LC. The 11.5 to 12.5 min fractions were collected, concentrated, and reinjected for a Q3 product ion scan. *A*, total ion chromatogram of the reinjected sample. *B*, Q3 mass scan of endogenous 9cRA. 9cRA, 9-*cis*-retinoic acid; LC, liquid chromatography.

To further confirm 9cRA authenticity, 46 pooled mouse pancreata were analyzed after methanol homogenization, followed by extraction with hexane. Endogenous 9cRA was resolved from other RA isomers by high-performance LC. Reinjection of the collected 9cRA fraction for a Q3 mass scan generated a mass spectrum consistent with that of a 9cRA standard (Fig. 3) (38). The protonated molecular ion occurred at *m/z* 301. Characteristic product ions were present at *m/z* 283, 255, 205, and 123. These ions identified the molecular weight as an RA, with loss of water, loss of the carbonyl group, presence of the side-chain plus a portion of the β-ionone ring and the β-ionone ring, respectively.

### Verification of 9cRA by derivatization with 3-NPH

**Figure 4:**
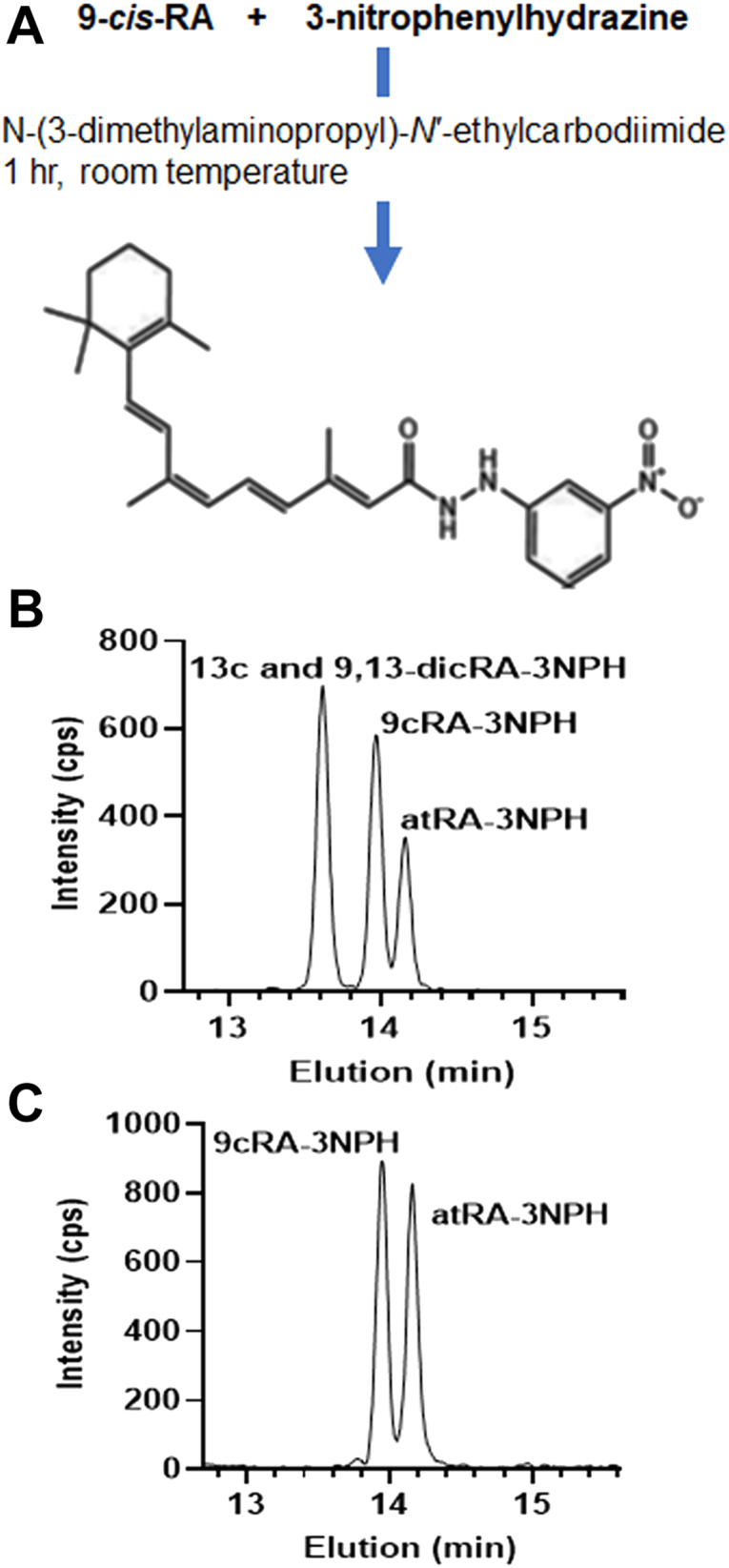
**Derivatization of endogenous 9cRA with 3-NPH.***A*, reaction of 9cRA with 3-NPH. *B*, chromatogram of 9,13dcRA-3-NPH, 9cRA-3-NPH and atRA-3-NPH standards (300 fmol each). *C*, livers from six mice were homogenized with methanol and analyzed by LC/MS/MS for comparison to authentic 3-NPH derivatives. The *Y*-axes shows intensity of the product ion in Q3 (m/z 283.3). 3-NPH, 3-nitropenylhydrazine; 9cRA, 9-*cis*-retinoic acid; LatRA, all-*trans*-retinoic acid; C/MS/MS, LC-tandem mass spectrometry.

Derivatization with 3-nitropenylhydrazine (3-NPH) provided another independent confirmation of 9cRA authenticity (Fig. 4*A*). First, we tested derivatizing atRA with 3-NPH using N-(3-dimethylaminopropryl)-N′-carbodiimide as catalyst. Mass transitions of atRA-3-NPH were determined by Q1 and Q3 scans (Fig. S1). Having validated the structure of the atRA 3-NPH derivative, six liver samples were homogenized individually with methanol and extracted with hexane. One of 40 μl was injected into the LC-atmospheric pressure chemical ionization (APCI)-MS/MS to confirm presence of 9cRA and atRA. The remaining 39 μl from each sample were pooled, concentrated, derivatized, extracted, and compared to RA 3-NPH standards. The endogenous 9cRA-3-NPH derivative comigrated with a synthetic 9cRA-3-NPH derivative and had identical mass characteristics through Q1 and Q3 (Fig. 4, *B* and *C*).

### Assay of tissue RA isomers

**Figure 5:**
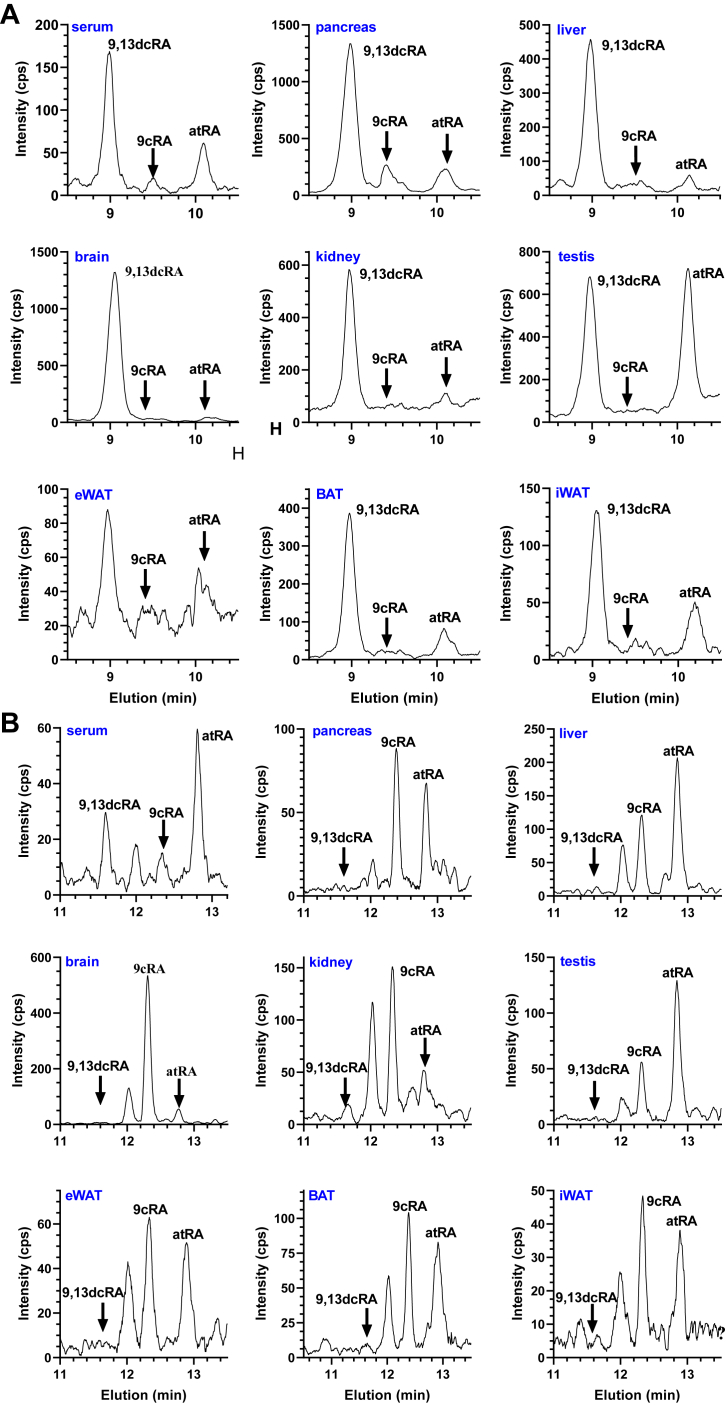
**Assay of tissue RA.***A*, mouse tissues were homogenized in saline/ethanol and analyzed by the original LC protocol. *B*, mouse tissues were homogenized in methanol and analyzed by the revised LC protocol. In both cases, detection was done by LC/MS/MS including comigration with authentic standards, selection of the molecular ion at *m/z* 301 in Q1, and quantification of the characteristic product ion at *m/z* 205 in Q3. *Y*-axes indicate intensity of the Q3 signal. LC, liquid chromatography; LC/MS/MS, LC-tandem mass spectrometry; RA, retinoic acid.

Both protocols were applied to mouse tissues to determine impact on the composition of RA isomers. Identification of RA isomers consisted of LC comigration with authentic standards, selection of the molecular ion at *m/z* 301 by Q1, and quantification of the characteristic product ion at *m/z* 205 in Q3. The ethanol protocol provided results equivalent to the original reports authenticating 9cRA in pancreas and presence of 9,13dcRA, but not 13cRA (Fig. 5*A*), and failing to observe 9cRA above the limits of quantification outside pancreas (17, 18). Serum and liver included atRA, relatively high concentrations of 9,13dcRA, and undetectable 13cRA, as reported originally. The brain showed a high level of 9,13dcRA, with atRA and 9cRA below limits of quantification. A screen of kidney, testis, epididymal white adipose tissue (eWAT), inguinal white adipose tissue (iWAT) and brown adipose tissue (BAT) revealed similar results. Comparatively, the methanol-based protocol revealed presence of 9cRA and atRA in the pancreas, but did not include 9,13dcRA or 13cRA (Fig. 5*B*). The methanol-based protocol showed 9,13dcRA only in serum, confirming a previous report (39). The revised protocol detected 9cRA in all other tissues assayed: the liver, the brain, the kidney, testes, eWAT, BAT, and iWAT, and did not show 9,13dcRA.

### Fasting affects 9cRA and atRA tissue concentrations independently

9cRA concentrations exceeded atRA in the brain, the kidney, eWAT and fasted pancreas (Table 1). atRA concentrations exceeded 9cRA in the liver and testis. 9cRA and atRA occurred at comparable levels in BAT and iWAT. A 16-h fast increased 9cRA 3-fold and atRA 2-fold in pancreas and >4-fold in eWAT. 9cRA levels also were increased by fasting in the BAT (1.7-fold) and brain (2.3-fold), whereas atRA levels were not affected. Fasting decreased 9cRA ∼50% in the kidney. Fasting increased atRA ∼1.9-fold in the liver, consistent with previous reports (40, 41, 42). Fasting/refeeding did not affect atRA levels in serum, BAT, iWAT, kidney, brain, and testis.

**Table 1:** Variations of 9cRA and atRA with fasting *versus* refeeding

Tissue (ng/mg protein)	9cRA		atRA	
	Fasted	refed	Fasted	refed
Serum (nM)	--	--	2.9 ± 0.4	2.9 ± 0.6
Pancreas	9.7 ± 0.6	3.1 ± 0.4 (*p* < 0.02)	6.4 ± 0.6	3.1 ± 0.4 (*p* < 0.002)
Liver	4.8 ± 0.4	4.1 ± 0.4	12.3 ± 1.3	6.6 ± 0.6 (*p* < 0.003)
Brain	34 ± 2.2	14.7 ± 1.3 (*p* < 0.0001)	4.0 ± 0.5	4.3 ± 0.7
Kidney	6.5 ± 1.1	13.5 ± 1.3 (*p* < 0.003)	2.2 ± 0.4	3.1 ± 0.4
Testes	2.8 ± 0.4	3.5 ± 0.5	7.3 ± 0.2	8.0 ± 0.7
eWAT	18.8 ± 1.4	4.3 ± 0.7 (*p* < 0.0001)	11.2 ± 1.2	2.5 ± 0.5 (*p* < 0.0001)
BAT	3.6 ± 0.3	2.1 ± 0.3 (*p* < 0.002)	2.8 ± 0.5	3.1 ± 0.5
iWAT	7.0 ± 0.9	7.0 ± 1.1	7.5 ± 0.9	7.8 ± 1.5

Mice were fasted 16 h or fasted 16 h and refed 6 h (n = 6, means ± SE). *p* values refer to the differences between fasted and refed values. Serum 9cRA samples were lost.

### Glucose reduces 9cRA biosynthesis

**Figure 6:**
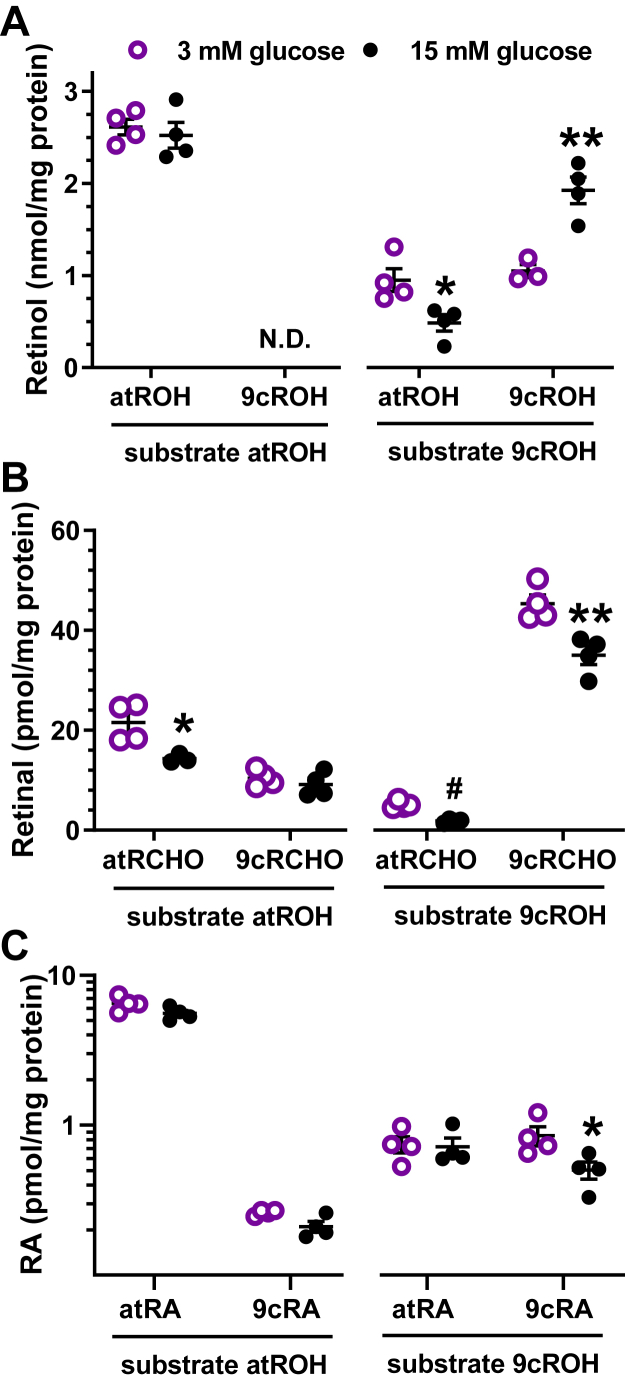
**9cRA biosynthesis in a β-cell line.** 832/13 cells were FBS starved 16 h in medium with 11.1 mM glucose and then incubated 4 h with 250 nM all-*trans*-retinol or 9-*cis*-retinol and 3 mM or 15 mM glucose with continuing FBS starvation. Retinoids were quantified by LC/MS/MS. *A*, retinol isomers recovered. *B*, retinal isomers generated. *C*, atRA and 9cRA generated: ∗*p* < 0.05, ∗∗*p* < 0.02, #*p* < 0.001 *versus* 3 mM glucose. 9cRA, 9-*cis*-retinoic acid; atRA, all-*trans*-retinoic acid; FBS, fetal bovine serum; LC/MS/MS, LC-tandem mass spectrometry.

Mouse models with depleted β-cell populations indicate that β-cells account for ∼70% of pancreas 9cRA. Moreover the β-cell model 832/13 produces 9cRA from 9-*cis*-retinol (17). GSIS in β-cells has been investigated extensively in 832/13 cells (43, 44, 45). We used 832/13 cells to determine whether glucose regulates 9cRA biosynthesis. We quantified retinol uptake and production of retinal and RA isomers in 832/13 cells treated with 250 nM all-*trans*-retinol or 9-*cis*-retinol (Fig. 6). Exposure to all-*trans*-retinol increased intracellular all-*trans*-retinol, but did not result in the presence of 9-*cis*-retinol (Fig. 6*A*). The medium glucose concentration did not affect the amount of intracellular all-*trans*-retinol. Cells treated with 9-*cis*-retinol had both all-*trans*-retinol and 9-*cis*-retinol present. Fifteen millimolar glucose decreased the amount of all-*trans*-retinol by 49%, whereas it increased 9-*cis*-retinol by 1.8-fold compared to the 3 mM glucose-treated group. All-*trans*-retinol generated mostly all-*trans*-retinal and about half as much 9-*cis*-retinal (Fig. 6*B*). In contrast, 9-*cis*-retinol generated predominantly 9-*cis*-retinal, with minor amounts of all-*trans*-retinal. Retinal concentrations with 3 mM glucose exceeded those of 15 mM glucose, with exception of 9-*cis*-retinal during all*-trans*-retinol treatment. Both atRA and 9cRA were detected with all-*trans*-retinol substrate, with much higher atRA than 9cRA (Fig. 6*C*). 9-*cis*-retinol generated similar levels of atRA and 9cRA. Fifteen millimolar glucose decreased 9cRA biosynthesis from 9-*cis*-retinol ∼40%.

### Glucose suppresses *Rdh5* transcription

**Figure 7:**
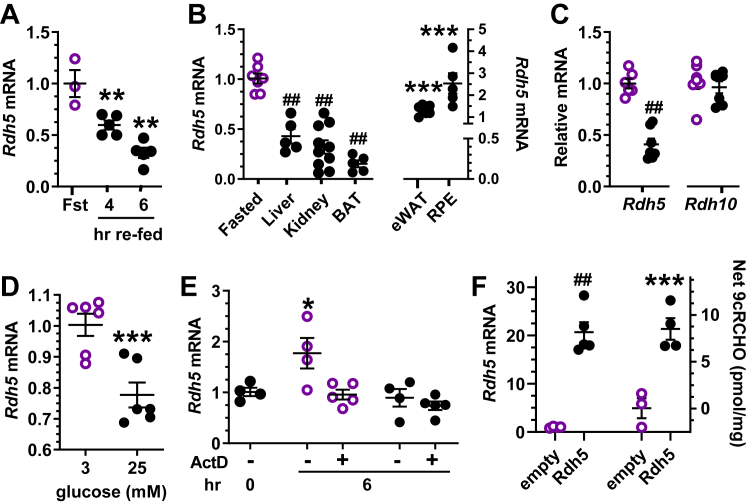
**Regulation of *Rdh5* mRNA.***A*, mouse pancreas *Rdh5* mRNA after 16 h fasting or after 6 h refeeding following a 16 h fast (3–5 mice per condition). *B*, *Rdh5* expression in mouse tissues after a 16 h fast, or after 6 h refeeding following a 16 h fast. *Open symbols*, 16 h fast. *Filled symbols*, 6 h refeeding following a 16 h fast. Data show a representative experiment of 1 to 4 (5–10 mice per condition; ∗∗∗*p* < 0.005, ##*p* < 0.0001 *versus* fasted. BAT, brown adipose tissue; eWAT, epididymal white adipose tissue; RPE, retinal pigment epithelium. *C*, glucose represses *Rdh5* mRNA in the 832/13 β-cell model. Cells were maintained 6 h in serum-free medium with 3 mM glucose (*open symbols*) or 15 mM glucose (*filled symbols*): ##*p* < 0.02 *versus* 3 mM glucose. *D*, effect of glucose on *Rdh5* mRNA in MIN6 cells. Cells were incubated 16 h in serum-free medium with 3 mM or 25 mM glucose: ∗∗∗*p* < 0.005 *versus* 3 mM glucose. *E*, 832/13 cells were incubated overnight in serum-free medium containing 15 mM glucose to decrease *Rdh5* mRNA (reference condition at 0 h); then incubated 6 h in serum-free medium containing 3 mM (*open symbols*) or 15 mM (*filled symbols*) glucose ±0.1 μg/ml actinomycin D. Data show a representative experiment of four with 4 to 5 replicates per condition: ∗*p* < 0.05 from 0 h group. *F*, 832/13 cells were transfected with 10 ng pSG5 Rdh5 or control vector and treated 24 h in serum-free medium containing 3 mM glucose, then 4 h in serum-free medium containing 3 mM glucose with 250 nM 9-*cis*-retinol. *Rdh5* mRNA and net 9-*cis*-retinal were normalized to cell protein: ∗∗∗*p* < 0.005, ##*p* < 0.0001 *versus* control vector.

Tissues express *Rdh5* widely and recombinant *Rdh5* catalyzes conversion of 9-*cis*-retinol into 9cRA efficiently (46). Decreases in 9-*cis*-retinal and 9cRA with 15 mM glucose suggest that refeeding *versus* fasting affects *Rdh5* mRNA or Rdh5 activity, as it does for *Rdh1* and *Rdh10* in the liver (40, 41, 42). Refeeding after a fast decreased *Rdh5* mRNA in pancreas, with reductions of 40 and 67% in 4 and 6 h, respectively (Fig. 7*A*). Reductions after refeeding were not limited to pancreas (Fig. 7*B*). Six hours after refeeding *Rdh5* mRNA decreased in the liver, kidney, and brown adipose tissue by 68, 93, and 92%, respectively, but increased 15% in eWAT and 80% in retinal pigment epithelium. Regulation of *Rdh5* mRNA by glucose was tested in 832/13 cells (Fig. 7*C*). Glucose (15 mM) reduced *Rdh5* mRNA ∼60% relative to 3 mM glucose after 6 h, without affecting *Rdh10*. We also evaluated the mouse pancreas cell line MIN6, another model of β-cell function (Fig. 7*D*) (47). *Rdh5* mRNA in the presence of 3 mM glucose exceeded that of 15 mM glucose by 1.8-fold. Actinomycin D (ActD) prevented the increase in *Rdh5* mRNA prompted by 3 mM glucose, indicating that low glucose permits activation of *Rdh5* transcription (Fig. 7*E*). ActD did not decrease *Rdh5* mRNA further than 15 mM glucose, indicating 15 mM glucose prevents *Rdh5* transcription. To confirm that Rdh5 catalyzes 9cRA biosynthesis, 832/13 cells were transfected with *Rdh5* (Fig. 7*F*)*. Rdh5* overexpressing cells increased *Rdh5* mRNA and net conversion of 9*-cis*-retinol into 9cRA.

### Glucose reduces 9cRA biosynthesis through Akt independent of insulin

**Figure 8:**
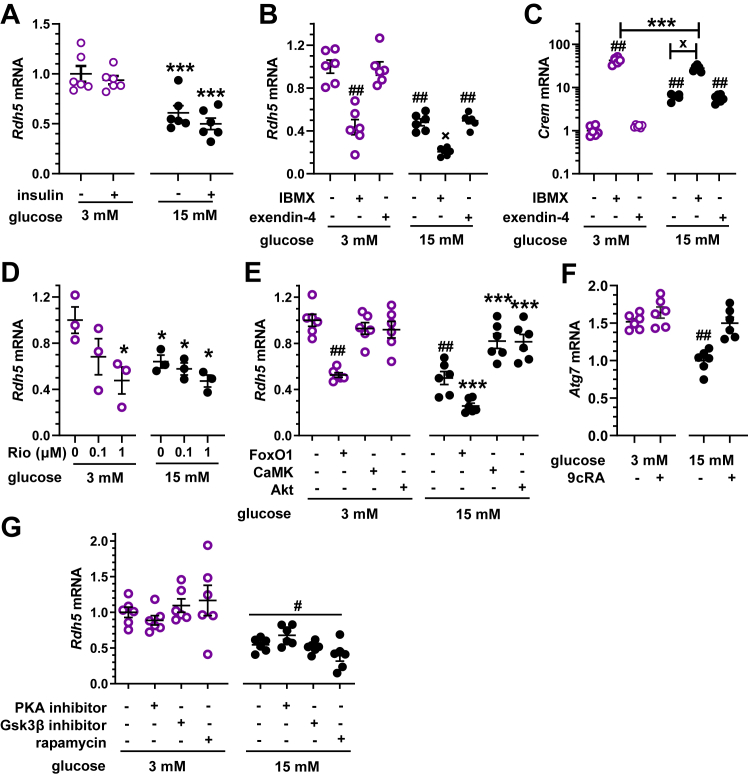
**Mechanism of glucose action.** β-cells (832/13) were incubated 6 h with serum-free medium containing 3 mM or 15 mM glucose plus the additions noted. *A*, insulin effects on *Rdh5* mRNA. Cells were incubated in the presence or absence of insulin (100 ng, n = 6 plates/group, ∗∗∗*p* < 0.005 *versus* 3 mM glucose. *B*, IBMX (10 μM) and exendin-4 (10 nM) effects on *Rdh5* mRNA (n = 6, ##*p* < 0.0001 *versus* 3 mM glucose; x*p* < 0.0001 *versus* 3 mM glucose and IBMX. *C*, IBMX (10 μM) and exendin-4 (10 nM) effects on *Crem* mRNA (##*p* < 0.0001 *versus* 3 mM glucose; ∗∗∗*p* < 0.005 *versus* 3 mM glucose with IBMX; x*p* <0.0001 *versus* 15 mM glucose. *D*, riociguat (Rio) effects on *Rdh5* mRNA (∗*p* < 0.05 *versus* 3 mM glucose). *E*, effects of FoxO1 (100 nM AS1842856), CaMK (1 μM CK59), or Akt (1 μM Triciribine hydrate) inhibitors (##*p* < 0.0001 *versus* 3 mM glucose, ∗∗∗*p* < 0.005 *versus* 15 mM glucose. *F*, effect of 9cRA on *Atg7* mRNA (##*p* < 0.0001 *versus* 3 mM glucose). 9cRA (1 nM) increases *Atg7* mRNA in 832/13 cells in the presence of 15 mM glucose (##*p* < 0.0001 *versus* 3 mM glucose). *G*, effects of inhibitors of mTOR (10 nM rapamycin), PKA (400 nM 14–22 amide) or GSK3β (100 nM XXV) (#*p*< 0.001 *versus* 3 mM glucose). 9cRA, 9-*cis*-retinoic acid; IBMX, 3-isobutyl-l-methylxanthine.

We tested insulin to determine the possibility of negative feedback on 9cRA biosynthesis (Fig. 8*A*). Insulin had no effect on *Rdh5* mRNA, regardless of the medium glucose concentration. We then applied pharmacological approaches to major aspects of glucose signaling to identify pathways linking glucose to *Rdh5* expression. Glucose increases cAMP in β-cells and 3-isobutyl-l-methylxanthine (IBMX) increases cAMP and cGMP (48). IBMX decreased *Rdh5* expression by 52 to 56% in 3 mM and 15 mM glucose, respectively (Fig. 8*B*). Glucagon-like peptide-1 (GLP-1) targets β-cells, among others, and increases β-cell cAMP (49). The GLP-1 analog exendin-4, however, had no effect on *Rdh5* mRNA, regardless of the glucose concentration. Increased glucose *via* cAMP activates *Crem* (cAMP response element modulator), which encodes transcription factors that regulate gene expression in β-cells (50). IBMX increased *Crem* mRNA by 46-fold and 5-fold with 3 mM or 15 mM glucose, respectively. This result does not support a contribution of *Crem*-encoded proteins to *Rdh5* regulation, because unlike *CREM*, IBMX affected *Rdh5* mRNA to the same extent despite the glucose concentration. Exendin-4 had no effect on *Crem* mRNA regardless of the glucose concentration, excluding GLP-1 effects. Because IBMX increases both cAMP and cGMP, we tested the impact of riociguat, a guanylate cyclase activator (Fig. 8*D*). Riociguat suppressed *Rdh5* transcription only in the presence of low glucose conditions, indicating that cGMP suppresses *Rdh5* transcription only during low glucose and confirming cAMP suppresses *Rdh5* transcription in the presence of high glucose. Next, we investigated signaling downstream of glucose-ATP-Ca^2+^. During low glucose, the FoxO1 inhibitor AS1842856 reduced *Rdh5* expression by ∼50%, that is, to the same extent as high glucose (Fig. 8*E*). With high glucose, the FoxO1 inhibitor suppressed *Rdh5* expression another 50%. These data indicate that FoxO1 serves as the proximate inducer of *Rdh5*. During low glucose, neither the CaMK inhibitor nor the Akt inhibitor affected *Rdh5* expression. In 15 mM glucose, CaMK and Akt inhibitors prevented *Rdh5 r*epression, indicating involvement of both in *Rdh5* transcription. This again indicates a difference in mechanism between low and high glucose. Inhibitors of PKA, GSK3β, or rapamycin had no effect on high glucose-mediated *Rdh5* repression (Fig. 8*G*). These data suggest that CaMK transmits glucose signaling through Akt and Foxo1 to *Rdh5*.

### 9cRA induces β-cell autophagy

Autophagy maintains β-cell mass and function (51). RAR induces autophagy by binding to a retinoic acid receptor response element in the *Atg7* promoter (52, 53). High glucose decreased *Atg7* mRNA relative to lower glucose (Fig. 8*F*). 9cRA restored *Atg7* mRNA during high glucose to that of low glucose levels.

## Discussion

This report establishes existence of 9cRA in multiple tissues at concentrations similar to or greater than atRA. Identification rested on multiple criteria including the following: (1) selective ion monitoring of multiple tissues relying on comigration of endogenous 9cRA with synthetic 9cRA during high-resolution LC, accompanied by selection of the molecular ion in Q1 at *m/z* 301 [M + H^+^], followed by verification of a characteristic product ion in Q3 at *m/z* 205; (2) a full-scan mass spectrum of endogenous 9cRA revealing the typical molecular ion and product ions after isolation by high-resolution LC; (3) derivatization and comigration of the endogenous 9cRA derivative with a synthetic 9cRA derivative during high-resolution LC. Each of these procedures alone would have authenticated the analyte as 9cRA. The three together should resolve unmistakably the presence of 9cRA in tissues. This work also resolved why 9cRA has not been observed previously in tissues other than pancreas. Apparently, a combination of ethanol/saline-based homogenization and tissue-specific matrix effects causes 9cRA to isomerize and/or to extract poorly, reducing it below limits of quantification. Methanol precipitates proteins more efficiently than the saline/ethanol mixture. Homogenization in methanol, with removal of precipitated proteins before hexane extraction, diminishes matrix effects and prevents isomerization.

atRA has been accepted as an autacoid with diverse actions, that is, an endogenous retinoid effector of diverse biological processes (54). Debate over the biological significance of atRA was resolved by demonstrating its occurrence in tissues (55) and the discovery of RAR (56, 57). 9cRA satisfies the criteria that established the significance of atRA, and therefore should experience acceptance as a retinoid-derived autacoid. The many therapeutic actions of 9cRA and rexinoids contribute to this realization (58). Dissimilar tissue-specific distributions of 9cRA and atRA, and their individual responses to fasting/refeeding cycles, indicates that they contribute independently to retinoid action, with potentially distinct activities.

Because of its catalytic activity with 9-*cis*-retinol, and its relatively high mRNA expression in pancreas, this work examined the possibility that Rdh5 participates in pancreas 9cRA biosynthesis. Rdh5 is better known as one of the 11-*cis*-retinol dehydrogenases important to the visual cycle (59, 60). Mutations in *Rdh5* cause the disease fundus albipunctatus which retards dark adaptation and results in night blindness (61, 62). Rdh5, however, has equivalent catalytic efficiency (*kcat*/*Km*) with 9-*cis*-retinol and has widespread mRNA expression (46). The current data connects Rdh5 with the first and rate-limiting step of 9cRA biosynthesis. This does not imply that Rdh5 represents the only Rdh that participates in 9cRA biosynthesis. Rdh1 and Rdh10, for example, have activity with *cis*-retinols (63) Moreover, 9-*cis*-carotenoids occur in nature and undergo metabolism into 9-*cis*-retinal, thereby providing a source of *cis*-retinoids (64). This report also confirms the reports that a glucose challenge reduces pancreas 9cRA. In addition to the nongenomic mechanism of decreasing 9cRA, glucose reduces *Rdh5* mRNA by inhibiting its transcription. Glucose, therefore, counteracts the biological functions of 9cRA by reducing its concentrations through both transcriptional and posttranscriptional mechanisms.

**Figure 9:**
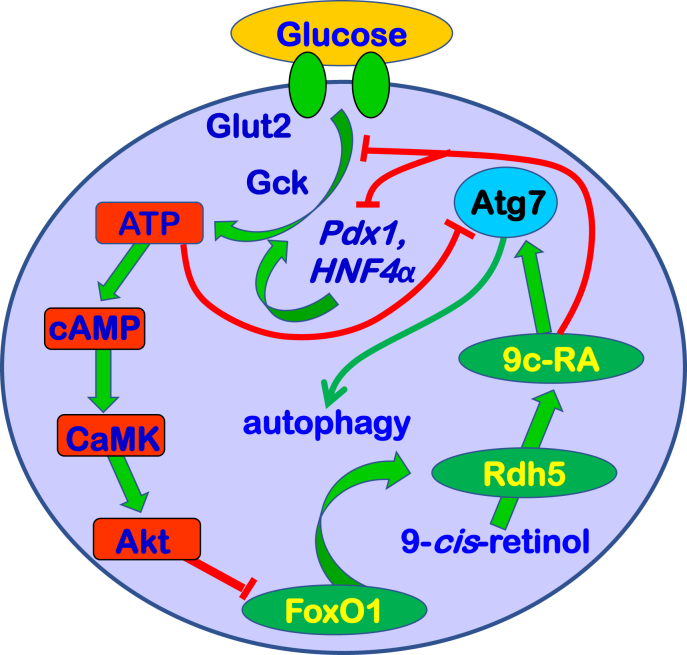
**Glucose and 9cRA exert opposing actions on β-cells.** The glucose transporter Glut2 enables glucose uptake by β-cells. Glucokinase (Gck) catalyzes phosphorylation of intracellular glucose, which undergoes glycolysis to generate pyruvate. Acetyl-CoA formed from pyruvate enters the mitochondria to produce ATP. The increase in ATP induces depolarization of the K^+^ channel, which prompts insulin release ((91)). Increased ATP also stimulates cAMP biosynthesis *via* adenylate cyclase (Adcy), which induces calmodulin-dependent protein kinase (CaMK). CamK activates Akt, which phosphorylates the transcription factor FoxO1, resulting in its expulsion from the nucleus. Glucose metabolism also results in the repression of the vital autophagy regulator *Atg7*. FoxO1 induces expression of *Rdh5*, which catalyzes the first and rate-limiting reaction in 9cRA biosynthesis. The current data and previous reports show that 9cRA exerts diverse actions on β-cells (17, 18). 9cRA rapidly reduces activities of Glut2 and Gck through nongenomic mechanisms. It represses transcription of *Pdx1* and *HNF4α*. The latter two transcription factors induce transcription of *Glut2* and *Gck*, decreasing glucose metabolism and thereby tempering GSIS. 9cRA also induces *Atg7*, maintaining β-cell autophagy and vigor. Even though Adcy, CaMK, and Akt affect the activities of PKA, mTOR, and GSK3β, the latter do not affect *Rdh5* mRNA. 9cRA, 9-*cis*-retinoic acid; GSIS, glucose-stimulated insulin secretion.

Chronic hyperglycemia creates glucotoxicity through raising ATP and cAMP levels, which impair GSIS and trigger β-cell dysfunction (65). cAMP induces *Crem*, which encodes inducible cAMP early repressor (ICER), a protein that reduces the insulin content of islets and contributes to impairing GSIS (50, 66, 67, 68). Ultimately, these and many other gene expression abnormalities set up β-cell failure and apoptosis. 9cRA counteracts glucotoxicity by posttranscriptionally reducing Glut2 and Gck activities, and transcriptionally repressing *Pdx1* and *HNF4α*, two transcription factors that induce *Glut2* and *Gck* expression (19, 20, 69, 70). Glucotoxicity also impairs β-cell function by preventing autophagy, a process essential for maintaining healthy β-cells (71, 72). 9cRA counteracts glucotoxicity by limiting uptake and metabolism of glucose and by inducing *Atg7*, the key enzyme essential for autophagy (Fig. 9).

The substantial concentration of 9cRA in brain relative to atRA, and its adjustment to fasting suggests a critical contribution to regulating feeding behavior and energy use. 9cRA at low nM concentrations affects expressions of multiple genes in human neural stem cells (73). Much less is known about 9cRA neural effects compared to atRA. But atRA actions could inform those of 9cRA. atRA exerts both posttranscriptional and transcriptional effects on neurite outgrowth, brain development, memory formation, and various neurological diseases, including fetal alcohol spectrum disorders, Alzheimer’s disease, Parkinson’s disease, and glaucoma (74, 75, 76, 77, 78, 79). Aberrant RAR and RXR signaling results in neuropathology, whereas atRA and rexinoids alleviate neuronal diseases (80, 81, 82). For both RAR and RXR, either defective signaling or overstimulation by pharmacological ligand concentrations cause pathology (83, 84). Therefore, it seems likely that 9cRA neurological effects would overlap with and exceed those of atRA. Future research should address these possibilities.

FoxO1 serves as the nexus between the counter effects of glucose *versus* 9cRA in β-cells. Insulin in liver phosphorylates Akt/Pkb, which phosphorylates FoxO1. Phosphorylated FoxO1 translocates from the nucleus to undergo cytosolic proteolysis. The absence of nuclear FoxO1 deactivates the RA biosynthetic pathway by reducing transcription of *Rdh1, Rdh10, Dhrs9, Raldh1, Raldh3, and Rbp1* (85). The current work shows that insulin does not affect β-cell 9cRA biosynthesis and *Rdh5* mRNA. Instead, glucose metabolism activates Akt independently of insulin to suppress FoxO1. FoxO1 has multiple beneficial effects on β-cells, such as reducing oxidative stress, regulating mitochondria-mediated metabolism, promoting autophagy, and reducing DNA damage, all key processes related to glucotoxicity (86). Activation of *Rdh5* transcription by FoxO1 indicates that supporting 9cRA biosynthesis involves another mechanism whereby FoxO1 promotes β-cell fitness.

Calléja *et al.* (87) excluded an RA serving as a universal ligand for RXRα partnered with PPARβ/δ or RARγ in epidermal suprabasal keratinocytes, in which RARγ transcriptionally represses unknown genes. Formation of lamellar granules in these skin cells requires transcriptional activation by liganded PPARβ/δ/RXRα and transcriptional repression by unliganded RARγ/RXRα, with RARγ dominating RXRα. They concluded that an RA binding to both RARγ and RXRα would produce defective lamellar granules by activating RARγ, Clearly, both RARγ and RXRα cannot undergo activation simultaneously in this context, but neither does RXRα activation seem obligatory. Therefore, explanations other than the one proposed emerge as plausible. For example, the authors noted that retinoids do not occur in mouse keratinocytes. This reveals a physiological mechanism to promote lamellar granule formation, Cyp26s have a well-known function to catabolize RAs to protect retinoid-sensitive locations (83). This established mechanism eclipses the nonphysiological interventions that fostered the conclusion. Suppression of RAR by alternative mechanism(s) might also occur. For example, the glucocorticoid receptor represses RA-induced transcription by interacting with the RAR/RXR transcription complex (41). Finally, with respect to a role for 9cRA as a signaling molecule, it has at least 5-fold higher affinity for RAR than RXR (88). This disparity introduces the possibility that 9cRA may prioritize RAR activation, depending on the tissue concentrations of each RA. This report establishes the presence of 9cRA as an endogenous retinoid in multiple tissues, in concentrations similar to or greater than atRA. Tissue concentrations of 9cRA vary with fasting and refeeding independently of atRA. Glucose decreases 9cRA concentrations and biosynthesis in pancreatic β-cells, whereas 9cRA counterbalances glucotoxicity. These data establish 9cRA as a retinoid-derived autacoid that moderates GSIS in pancreas, promotes β-cell vigor, and likely affects energy use in general.

## Experimental procedures

All aspects of these experiments were performed under yellow lights to prevent retinoid isomerization.

### Materials

Optima LC/MS grade methanol, acetonitrile, water, and formic acid were purchased from Thermo Fisher Scientific. Standards of atRA, 9cRA, 13cRA, and 3-nitrophenylhydrazine hydrochloride (3-NPH·HCl) and N-(3-dimethylaminopropyl)-N′-ethylcarbodiimide hydrochloride (EDC·HCl) were purchased from Sigma-Aldrich. Standard 9,13-di-*cis*-RA was purchased from Santa Cruz Biotechnology.

### Animal experiments

C57BL/6J (strain# 000664) mice were purchased from The Jackson Laboratory. Upon arrival, mice were fed a purified AIN93G diet (Research Diets, D10012G) containing 4 IU retinyl acetate/g and 7% fat and bred in-house through >3 generations. Blood, liver, pancreas, kidney, testis, epididymal and inguinal white adipose tissues (eWAT and iWAT), brown adipose tissue (BAT), and brain samples were collected from male 8∼9 weeks-old-mice, fasted 16 h and compared to those refed 6 h after a 16 h fast. Mice were euthanized in an isoflurane chamber, followed by cervical dislocation. Blood samples, drawn from the vena cava, were allowed to clot on ice and centrifuged 30 min at 12,000*g* and 4 °C. Sera and tissues were snap-frozen in liquid nitrogen and stored at −80 °C until assayed. Animal experimental protocols were approved by the University of California Berkeley Animal Care and Use Committee.

### Saline/ethanol homogenization

Tissues (50∼150 mg) or sera (100 μl) were homogenized in ice-cold 0.9% saline using a glass pestle tissue grinder (PYREX, 77245) with a motorized homogenizer (Heidolph, RZR1) at 280 rpm to produce a 10% homogenate. The homogenate (0.5 ml) was transferred to a 23 ml round-bottom glass tube and mixed with 10 μl internal standard (100 nM 3,3-dimethyl-RA in acetonitrile) and 3 ml of 25 mM KOH, prepared in ethanol, by vortexing for 10 s. Ten milliliters hexane was added. The mixture was vortexed 20 s and centrifuged 2 min at 2000*g*. The upper hexane phase was discarded. One hundred eighty microliters of 4 M HCl were added to the remaining aqueous phase. RAs were extracted with 10 ml hexane by vortexing 20 s. The upper hexane phase was transferred to a new round bottom glass tube and evaporated under a gentle stream of nitrogen in a water bath (Organomation Associates, N-EVAP 112) at 25 to 30 °C. The residue was reconstituted in 40 μl acetonitrile with 20 s vortexing, spun for 10 s at 1200*g*, and transferred to a glass vial insert. One microliter was injected for LC-APCI-MS/MS analysis.

### Methanol homogenization

Tissue samples (50∼150 mg) or sera (100 μl) were placed into a glass pestle tissue grinder with 1.5 ml of ice-cold methanol and 10 μl of the 100 nM internal standard, prepared in acetonitrile. Samples were homogenized using the motorized homogenizer at 280 rpm. The homogenate was transferred to a 15 ml glass conical tube (PYREX, 8060) and centrifuged 2 min at 1200*g* at room temperature. The supernatant was transferred to a clean glass round-bottom tube and mixed with 100 μl of 750 mM KOH, prepared in methanol, and vortexed 10 s. To remove hydrophobic impurities, 10 ml of hexane was added, vortexed 10 s, and spun 10 s at 1200*g*. The upper hexane layer was discarded. The bottom methanol phase was mixed with 180 μl 4 M HCl by vortexing 10 s and extracted with 10 ml hexane. The mixture was centrifuged 2 min at 1200*g*. The upper hexane layer was transferred to a new round-bottom glass tube and evaporated under a gentle stream of nitrogen with heating at 25 to 30 °C in a water bath. The residue was reconstituted in 40 μl of acetonitrile with 20 s vortexing, spun 10 s at 1200*g*, and transferred to a glass vial insert. One microliter was injected for LC-APCI-MS/MS analysis.

### Original LC conditions

Chromatography was done with an Agilent 1290 series liquid chromatograph consisting of a binary pump, temperature-controlled column compartment, and temperature-controlled autosampler. The column compartment and autosampler were maintained at 40 °C and 10 °C, respectively. The mobile phase included two components: component A (0.1% formic acid in water) and B (0.1% formic acid in acetonitrile). The following gradient was used: 0 to 3 min, hold at 70% B; 3 to 15 min, 70% B to 95% B; 15 to 20 min, hold at 95% B; 20 to 21 min, 95% B to 70% B; 21 to 25 min, re-equilibrate at 70% B. An Ascentis RP-Amide column resolved analytes (150 × 2.1 mm, 3 μm, Supelco 565302-U) fitted with a preceding guard column (Supelguard ABZ + PLUS, 20 × 2.1 mm, 5 μm, Supelco 59605), at a flow rate of 400 μl/min.

### Revised LC conditions

Resolution of RA isomers was achieved with an analytical Ascentis Express RP-Amide column (100 × 2.1 mm, 2.7 μm, Sigma-Aldrich, 53913-U) fitted with a preceding guard column at a flow rate of 500 μl/min. The following were the mobile phases used: (A) 0.1% formic acid in water/methanol (6:4); (B) 0.1% formic acid in acetonitrile/methanol (6:4). A gradient was applied over 25 min: 0 to 2 min, 40% B; 2 to 10 min, 40 to 55% B; 10 to 18 min, 55 to 95%; 18 to 21 min, holding at 95% B; 21 to 23 min, 95 to 40% B; 23 to 25 min, holding at 40% B.

### Tandem mass spectrometry (MS/MS)

Quantitative data were obtained using a Sciex API-4000 triple-quadrupole mass spectrometer, equipped with an APCI source, operated in positive ion mode. The instrument was controlled by Analyst v1.4 software (https://sciex.com/products/software/analyst-software) and operated in multiple reaction–monitoring mode. RAs were monitored using a m/z 301.1 [M + H]+ to m/z 205.0 transition. The internal standard 3,3-dimethyl-RA was monitored using a m/z 329.4 [M + H]+ to m/z 151.3 transition. The dwell time for both RA and 3,3-dimethyl-RA was 150 ms. Optimum positive APCI conditions included the following: collision gas, 7 psig; curtain gas, 10 psig; gas1, 70 psig; nebulizer current, 3 μA; source temperature, 350 °C; declustering potential, 55 V; entrance potential, 10 V; collision energy, 17 eV; and collision exit potential, 5 V.

### RA derivatization with 3-NPH

Standards of RA isomers (30 pmol each) were prepared in 50 μl acetonitrile. Remaining samples after the injection for RA extraction/quantification were pooled, evaporated under a gentle stream of nitrogen, and reconstituted in 50 μl acetonitrile. Twenty millimolar of 3-NPH was prepared in 40 mM HCl and ethanol (v/v 3:1). One hundred millimolar of EDC was prepared in pyridine and acetonitrile (v/v 1:1). In a 1.8 ml amber glass vial, 50 μl of sample was mixed with 100 μl 3-NPH and 75 μl EDC by vortexing 10 s, then incubated 1 h at room temperature. The reaction mixture was transferred to a 23 ml round-bottom glass tube and extracted with 10 ml hexane as described in the revised extraction section.

### LC conditions for RA-3-NPH

Resolution of RA-3-NPH isomers was achieved with the analytical Ascentis Express RP-Amide column with the preceding guard column at a flow rate of 400 μl/min. The following were the mobile phases used: (A) 0.1% formic acid in water; (B) 0.1% formic acid in methanol. The following gradient was applied over 25 min: 0 to 21 min, 30 to 85% B; 21 to 22.5 min, holding at 85% B; 22.5 to 23.5 min, 85 to 40% B; 23.5 to 25 min, holding at 30% B.

### MS/MS conditions for RA-3-NPH

RA-3-NPH was monitored using an m/z 436.7 [M + H]+ to m/z 283.3 transition. Positive APCI conditions included the following: collision gas, 7 psig; curtain gas, 10 psig; gas1, 70 psig; nebulizer current, 1 μA; source temperature, 350 °C; declustering potential, 55 V; entrance potential, 10 V; collision energy, 20 eV; collision exit potential, 5 V.

### Quantification of retinol and retinal

Retinol isomers were measured by HPLC/ultraviolet absorbance detection (89). Retinal was quantified using a published method (90) with LC modifications for improved resolution of retinal isomers: retinal-*O*-ethyloxime isomers were resolved *via* an Acclaim C30 HPLC Column (100 × 2.1 mm, 3 μm, Thermo Fisher Scientific, 078665) with a flow rate of 400 μl/min (mobile phase A: 0.1% formic acid in water, B: 0.1% formic acid in methanol) at a gradient: 0 to 2 min, hold at 80% B; 2 to 2.5 min, 80% B to 85% B; 2.5 to 17.5 min, 85% B to 95% B; 17.5 to 20 min, hold at 95% B; 20 to 20.5 min, 95% B to 80% B; 20.5 to 25 min, re-equilibrate at 80% B at 40 °C.

### Cell culture

The rat insulinoma cell line 832/13 was a gift from the Newgard lab (43). Cells were maintained in growth medium (RPMI 1640 containing 11.1 mM glucose, 10% heat-inactivated fetal bovine serum (FBS) (Gibco, 10082), 2 mM L-glutamine, 1 mM sodium pyruvate, 50 μM 2-mercaptoethanol, 100 U/ml penicillin, 100 μg/ml streptomycin and 10 mM Hepes) under 5% CO_2_ in T75 flasks or 6-well plates. To minimize effects of unknown factors, cells were cultured in FBS-free growth medium containing 11.1 mM glucose 18 h prior to experiments. MIN6 cells were maintained in growth medium (Dulbecco's modified Eagle's medium containing 4.5 g/l glucose) under 5% CO_2_ in T75 flasks or 6-well plates.

### RNA isolation and qPCR

RNA was isolated from tissues and cells with TRI reagent (Sigma-Aldrich, T9424) following manufacturer’s instructions. Complementary DNA (cDNA) was reverse-transcribed using iScript cDNA Synthesis Kit (Bio-Rad, 1708891). Real-time quantitative PCR (qPCR) was prepared with PrimeTime Gene Expression Master Mix (IDT, 1055772) and qPCR probes (mouse *Actb*, Mm00607939_s1; mouse *Rdh5*, Mm00506111_m1; rat *Rdh5*, Rn.PT.56a.11212766.g; rat *Gusb*, Rn.PT.39a.22214822.g, rat *Rdh10*, Rn00710727_m1; rat *Crem*, Rn01538528_m1) and run on CFX Connect Real Time System (Bio-Rad). Gene expression was analyzed by the *ΔΔ*-Ct method, normalized to *Actb* (mouse) or *Gusb* (rat), and expressed as fold-change relative to expression in reference condition.

### Inhibitor assays

Inhibitors were purchased from: FoxO1, AS1842856 (EMD Millipore, 344355); GSk3β, XXV (EMD Millipore, 361568); PKA, 14 to 22 Amide (Sigma-Aldrich, 476485); Akt, triciribine hydrate (Sigma-Aldrich, T3830); mTOR, rapamycin (Sigma-Aldrich, R8781); CaMK, CK59 (Sigma-Aldrich, 208922). 832/13 cells were seeded on 6-well plates and maintained in growth medium. FBS-starved cells were treated with serum-free growth medium containing 3 mM or 15 mM glucose ± inhibitors for 6 h, followed by RNA isolation, cDNA synthesis, and qPCR.

### Data presentation and statistics

Data are expressed as mean ± SEM. Statistical analyses were made by two-tailed unpaired t tests using GraphPad Prism ver. 9.5.1.

## Data availability

All data are present in this manuscript.

## Supporting information

This article contains supporting information.

## Conflict of interest

The authors declare that they have no conflicts of interest with the contents of this article.
