# Supplementary material for: Energy status regulates levels of the RAR/RXR ligand 9-cis-retinoic acid in mammalian tissues: Glucose reduces its synthesis in β-cells
Source: J Biol Chem. 2023 Sep 14;299(10):105255. doi: 10.1016/j.jbc.2023.105255 (PMC10582780; doi:10.1016/j.jbc.2023.105255)
Supplement: Supporting Figure S1 [file mmc1.docx]

**Supporting Information**

**Energy status regulates levels of the RAR and RXR ligand 9-*cis*-retinoic acid in mammalian tissues: glucose reduces its synthesis in β-cells**

Hong Sik Yoo, Kristin Obrochta. Moss, Michael A. Cockrum, Wonsik Woo, Joseph L. Napoli*

**Supporting Figure 1. Mass transitions of atRA-3-NPH.** (A) atRA-3-NPH, indicated by the

arrow, eluted at 12.7 min, as shown by the total ion chromatogram of the Q1 scan. (B) The *m/z* value of the peak was 436.7. (C) Total ion chromatogram of a Q3 product scan of atRA-3-NPH. (D) Q3 product mass scan.
